# An In Vitro Comparison of Dentinal Tubule Occlusion by Sodium Fluoride and Bioactive Glass Varnishes With and Without Erbium:Yttrium-Aluminum-Garnet (Er:YAG) Laser Activation

**DOI:** 10.7759/cureus.106540

**Published:** 2026-04-06

**Authors:** Tasmiya M Bhavikatti, Biji Brigit, Kiran Kumar Neelakantappa, Vandana Kumari, Parvati S Bangarimath

**Affiliations:** 1 Department of Conservative Dentistry and Endodontics, Government Dental College and Research Institute Bangalore, Bengaluru, IND

**Keywords:** bioactive glass, dentinal hypersensitivity, dentinal tubule occlusion, desensitizing agents, erbium:yag laser, laser activation, scanning electron microscopy, sodium fluoride varnish

## Abstract

Background

Laser irradiation effectively occludes dentinal tubules, reducing dentine hypersensitivity and improving restorative outcomes. Sodium fluoride varnish is widely used for dentinal sensitivity, while bioactive glass varnish promotes mineral deposition. The Erbium:Yttrium-Aluminum-Garnet (Er:YAG) laser, due to its high absorption by water and hydroxyapatite, may enhance the occlusive efficacy of these desensitizing agents.

Objective

This study aimed to evaluate the effect of Er:YAG laser activation on the dentinal tubule-occluding efficacy of sodium fluoride and bioactive glass varnishes.

Methods

Sixteen extracted premolars were sectioned to yield 32 dentinal discs and divided into two main groups: sodium fluoride (Poco) and bioactive glass (Prevest). Each group was further subdivided into varnish application without laser activation and varnish application with Er:YAG laser activation. The mean number of open dentinal tubules was evaluated using scanning electron microscopy (SEM). Data were analyzed using IBM SPSS Statistics for Windows, Version 26 (released 2018; IBM Corp., Armonk, NY, USA). Normality was assessed using the Shapiro-Wilk test, followed by one-way analysis of variance (ANOVA) and Tukey’s post hoc test at a 95% confidence level.

Results

A statistically significant difference was observed among the four groups (p < 0.05), primarily due to the laser-activated bioactive glass varnish, which showed the greatest dentinal tubule occlusion. Laser-activated sodium fluoride varnish and the non-laser groups showed intermediate tubule occlusion, with sodium fluoride without laser having the highest number of open tubules.

Conclusions

Er:YAG laser activation significantly enhances the tubule-occluding efficacy of both varnishes. Laser-activated bioactive glass varnish demonstrated the highest effectiveness, suggesting a potential utility in reducing dentinal hypersensitivity.

## Introduction

Dentinal hypersensitivity is a prevalent clinical problem, characterized by a short, sharp pain arising from exposed dentin in response to thermal, evaporative, tactile, osmotic, or chemical stimuli, which cannot be attributed to any other dental defect or pathology [1]. The most widely accepted explanation for this phenomenon is Brännström’s hydrodynamic theory, which attributes pain to the rapid movement of fluid within patent dentinal tubules, stimulating mechanoreceptors at the pulp-dentin interface [2]. Consequently, the fundamental principle in managing dentinal hypersensitivity is to achieve effective and long-lasting occlusion of dentinal tubules, thereby reducing fluid movement and neural stimulation [3,4].

Sodium fluoride varnish has long been considered the gold standard for desensitization. Its mechanism involves the formation of calcium fluoride-like precipitates on the dentin surface and within the tubules, providing a mechanical barrier that reduces dentinal permeability [5,6]. However, these precipitates are prone to dissolution in acidic environments or under mechanical wear, limiting their long-term efficacy [7]. Bioactive glass-based varnishes have emerged as promising alternatives because of their bioactive and remineralizing potential. Upon contact with saliva or dentinal fluid, bioactive glass releases calcium, phosphate, and sodium ions, which increase the local pH and promote the formation of a hydroxycarbonate apatite (HCA) layer, chemically bonded to the dentin surface [8-10]. This leads to durable tubule occlusion and greater acid resistance compared to fluoride-based agents [11]. However, despite these advantages, certain studies have reported that bioactive glass particles may not always achieve uniform or complete penetration into deeper dentinal tubules, due to surface tension and particle aggregation [12]. Moreover, the rate of apatite formation can be relatively slow, which may delay the onset of desensitization [13].

The Erbium:Yttrium-Aluminum-Garnet (Er:YAG) laser (wavelength = 2940 nm) has been shown to effectively modify the dentin surface because of its high absorption in water and hydroxyapatite [14]. Laser irradiation can melt and resolidify peritubular dentin, resulting in narrowing or sealing of the tubules, while also creating a micro-roughened surface that enhances adhesion and mechanical interlocking of subsequently applied agents [15,16]. Studies have shown that combining Er:YAG laser treatment with desensitizing agents, such as varnishes, enhances their penetration and retention within dentinal tubules, accelerates mineral precipitation, and increases resistance to acid challenges [17-19]. Specifically, when bioactive glass varnish is laser-activated, the localized increase in surface temperature and microstructural alteration of dentin enhance ion exchange and apatite crystallization, thereby improving the uniformity and depth of tubule occlusion compared to the varnish used alone [20].

Therefore, laser activation of bioactive glass varnish is expected to overcome its inherent limitations, such as slow reaction kinetics and incomplete tubule coverage, leading to a more stable and durable occluding layer [21]. Despite these promising approaches, there is limited comparative evidence evaluating the synergistic effect of Er:YAG laser activation on sodium fluoride and bioactive glass varnishes.

Effective management of dentinal hypersensitivity is clinically important, as it significantly impacts patient comfort and quality of life, and successful dentinal tubule occlusion plays a key role in long-term symptom relief.

Hence, the present in vitro study aimed to compare the dentinal tubule-occluding efficacy of sodium fluoride varnish and bioactive glass varnish, with and without Er:YAG laser activation, using scanning electron microscopy (SEM). The null hypothesis was that there would be no significant difference in dentinal tubule occlusion among the experimental groups.

## Materials and methods

Specimen collection and preparation

Sixteen premolars, free from caries, restorations, fractures, or cracks, were collected from the Department of Conservative Dentistry and Endodontics, following extraction for orthodontic or therapeutic reasons. Teeth were cleaned of soft tissue and calculus using ultrasonic scalers and stored in 0.1% thymol solution at room temperature until use. From the cervical region of each root, two dentin slices, measuring approximately 5 × 5 × 3 mm, were prepared per tooth, yielding a total of 32 specimens, with the buccal surfaces oriented upward to expose dentin for treatment. Sectioning was performed using a low-speed diamond disc under copious water coolant to prevent overheating and structural damage. Each specimen was mounted in an acrylic resin block for stability and handling. Specimens were rinsed with distilled water and stored in artificial saliva at 37°C, to maintain dentinal moisture and simulate oral conditions.

Smear layer removal

Each specimen was immersed in 17% ethylenediaminetetraacetic acid (EDTA) for one minute to remove the smear layer, followed by rinsing with distilled water for 30 seconds, and gentle air-drying prior to varnish application. This duration was chosen to effectively expose dentinal tubules without causing excessive dentin erosion [22].

Sample size and grouping

A priori power analysis (G*Power version 3.1.9.7; Heinrich-Heine-Universität Düsseldorf, Düsseldorf, Germany) determined that 32 specimens would provide 90% power at an alpha level of 0.05 with an effect size of 1.0. Specimens were randomly allocated into two main groups (n = 16 each) based on the varnish used: sodium fluoride varnish (Poco; 5% NaF, approximately 22,600 ppm fluoride) and bioactive glass-based varnish (Prevest DenPro; containing calcium sodium phosphosilicate particles). Each group was further subdivided into two subgroups (n = 8 each) according to laser activation: (i) sodium fluoride varnish without laser activation, (ii) sodium fluoride varnish with Er:YAG laser activation, (iii) bioactive glass-based varnish without laser activation, and (iv) bioactive glass-based varnish with Er:YAG laser activation.

Varnish application

For the non-laser groups, varnishes were applied uniformly to the dentin surface using a sterile microbrush. A thin, even layer was achieved by brushing in a circular motion for approximately 10 seconds. Specimens were left undisturbed for the manufacturer-recommended setting time: Poco fluoride varnish, four to six hours; Prevest bioactive glass varnish, five minutes. After the setting time, specimens were lightly rinsed with distilled water and air-dried with oil-free, compressed air.

Laser activation parameters

For the laser-activated groups, specimens were irradiated after varnish application using an Er:YAG laser (wavelength = 2940 nm) in non-contact mode. Laser settings were 50 mJ per pulse, 30 Hz, 1.5 W, for 10 seconds, maintaining a 1-mm distance from the dentin surface. The handpiece was held perpendicular and moved in a sweeping motion for uniform energy distribution, preventing localized heating. All procedures were performed by a single, trained operator.

Specimen storage prior to analysis

Following treatment, specimens were rinsed with distilled water, air-dried with oil-free compressed air, and stored in artificial saliva at 37°C until SEM analysis.

SEM preparation and imaging

Specimens were mounted on aluminum stubs using double-sided conductive carbon tape, with the treated surface facing upward, and were sputter-coated with gold under vacuum. SEM examination was performed at Poornayu Research Lab, Bangalore, using a JEOL scanning electron microscope at 15 kV, capturing representative micrographs at 2000× magnification from the central region of each specimen. All imaging was conducted under standardized conditions by the same operator.

Quantitative analysis of dentinal tubules

SEM images were analyzed using Fiji (ImageJ, National Institutes of Health, Bethesda, MD, USA). A standardized region of interest (ROI) of 500 × 500 µm was defined at the center of each specimen. Open dentinal tubules were manually counted by a blinded and calibrated examiner. Three non-overlapping fields per specimen were analyzed, and the mean count per specimen was calculated. Intra-examiner reliability was assessed with an intraclass correlation coefficient (ICC) of 0.92.

Statistical analysis

Data were analyzed using IBM SPSS Statistics for Windows, Version 26 (released 2018; IBM Corp., Armonk, NY, USA). The level of statistical significance was set at p < 0.05. Descriptive statistics were calculated to determine the mean and standard deviation for each group. The normality of data distribution was assessed using the Shapiro-Wilk test. Since the data followed a normal distribution, inferential statistics were applied using one-way analysis of variance (ANOVA) to determine differences among the groups. Post hoc pairwise comparisons were performed using Tukey’s honestly significant difference (HSD) test. A 95% confidence level was considered for all statistical analyses.

## Results

The number of open dentinal tubules was evaluated in four experimental groups to assess the effectiveness of sodium fluoride and bioglass varnishes, with and without laser activation, in achieving dentinal tubule occlusion. Descriptive statistical analyses were performed to determine the mean, standard deviation, and 95% confidence interval of tubule counts in each group. The results are presented in Table 1.

**Table 1:** Descriptive statistics showing the number of open dentinal tubules in each experimental group This table presents descriptive statistics, including mean, standard deviation, standard error, and confidence intervals, for the number of open dentinal tubules observed in each experimental group. ANOVA: analysis of variance

	N	Mean	SD	Std. Error	95% Confidence Interval for Mean		Min	Max
					Lower Bound	Upper Bound		
Sodium fluoride	8	138.13	8.24	13.52	106.15	170.10	66	187
Laser-activated sodium fluoride	8	102.00	4.90	15.87	64.46	139.54	35	161
Bioglass	8	124.13	5.39	12.51	94.53	153.72	49	156
Laser-activated bioglass varnish	8	31.50	4.92	7.04	14.85	48.15	9	56
F-value (one-way ANOVA test)					13.99			

The mean number of open dentinal tubules observed in each group is presented in Table 1. The laser-activated bioglass varnish group (Group 2B) demonstrated the lowest mean number of open tubules (31.50 ± 4.92), indicating the highest degree of dentinal tubule occlusion among all experimental groups. In contrast, the sodium fluoride varnish group (Group 1A) exhibited the highest mean number of tubules (138.13 ± 8.24), representing the least effective occlusion. The laser-activated sodium fluoride varnish (Group 1B) and bioglass varnish (Group 2A) groups showed intermediate mean values of 102.00 ± 4.90 and 124.13 ± 5.39, respectively.

To determine whether significant differences existed between the experimental groups, pairwise comparisons were performed using the Tukey post hoc test following one-way ANOVA. The results of the pairwise comparisons between the groups are presented in Table 2.

**Table 2:** Post hoc Tukey test showing pairwise comparison of the number of open dentinal tubules between study groups Post hoc Tukey test was used for pairwise comparison of dentinal tubule counts between groups. * Indicates a statistically significant difference (p < 0.05).

Study Group	Study Group	Mean Difference	Std. Error	Sig. (p)	95% Confidence Interval
Sodium fluoride	Laser-activated SF	36.13	7.90	0.32	-14.70 - 86.95
Sodium fluoride	Bioglass	14.00	7.90	1.00	-36.83 - 64.83
Sodium fluoride	Laser-activated bioglass	106.63*	7.90	0.0001*	55.80 - 157.45
Laser-activated SF	Bioglass	-22.13	7.90	1.00	-72.95 - 28.70
Laser-activated SF	Laser-activated bioglass	70.50*	7.90	0.003*	19.67 - 121.33
Bioglass	Laser-activated bioglass	92.63*	7.90	0.0001*	41.80 - 143.45

**Figure 1:**
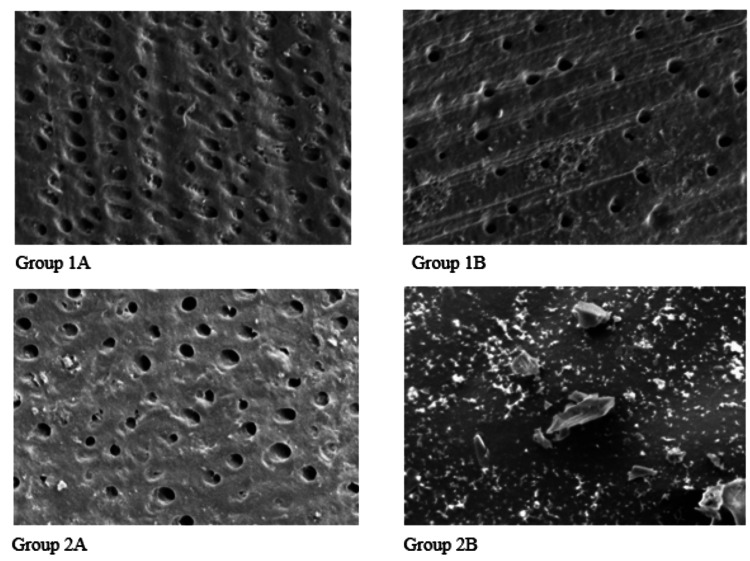
Scanning electron microscopy images showing dentinal tubule occlusion in different experimental groups Scanning electron microscopy images showing dentinal tubule occlusion in the experimental groups: (1A) sodium fluoride varnish, (1B) laser-activated sodium fluoride varnish, (2A) bioglass varnish, and (2B) laser-activated bioglass varnish.

The SEM analysis further demonstrated varying degrees of dentinal tubule occlusion among the experimental groups (Figure 1).

## Discussion

The present in vitro study evaluated the dentinal tubule-occluding efficacy of sodium fluoride and bioactive glass varnishes, with and without Er:YAG laser activation, using SEM and quantitative image analysis. One-way ANOVA demonstrated a statistically significant difference among the groups (p < 0.05). Post hoc Tukey analysis revealed that the laser-activated bioactive glass varnish group exhibited the greatest reduction in the number of open dentinal tubules, indicating superior occluding efficacy compared to the other groups.

The enhanced performance of laser-activated bioactive glass varnish can be attributed to the synergistic effect of its inherent bioactivity and laser-induced surface modification. Bioactive glass interacts with dentinal fluid by releasing calcium, phosphate, and sodium ions, leading to the formation of an HCA layer that closely resembles natural tooth mineral and provides durable tubule occlusion [23]. Previous studies have demonstrated that bioactive glass promotes remineralization and effectively occludes dentinal tubules through apatite deposition [24]. Furthermore, its efficacy depends on direct interaction with exposed dentinal tubules, facilitating ionic exchange and crystal nucleation [25].

In the present study, bioactive glass varnish without laser activation showed comparatively less tubule occlusion, which may be attributed to the limited penetration of particles into the dentinal tubules. Surface modification plays a crucial role in enhancing material interaction with dentin, and inadequate surface preparation may result in superficial deposition and incomplete sealing.

Er:YAG laser irradiation significantly improved the performance of both varnishes, particularly bioactive glass. Due to its wavelength of 2940 nm, the laser is highly absorbed by water and hydroxyapatite, allowing effective modification of the dentin surface without causing thermal damage [26]. Laser treatment facilitates smear layer removal, exposes dentinal tubules, and creates a micro-roughened surface that enhances wettability and promotes better penetration and retention of desensitizing agents [27]. These effects contribute to the improved tubule occlusion observed in the laser-activated groups.

The sodium fluoride varnish groups demonstrated comparatively higher numbers of open dentinal tubules. Fluoride varnishes act by forming calcium fluoride-like precipitates that provide temporary tubule occlusion; however, these deposits are loosely bound and susceptible to dissolution under acidic or mechanical conditions, limiting their long-term effectiveness [28]. Although laser activation slightly improved the performance of sodium fluoride varnish, the difference was not statistically significant, likely due to the superficial nature of fluoride deposits.

SEM analysis enabled detailed visualization of dentinal surface morphology and tubule occlusion and remains the gold standard for such evaluation. The use of Fiji (ImageJ) software further enhanced the accuracy and reproducibility of tubule quantification by minimizing subjective bias [29].

Despite these findings, certain limitations must be acknowledged. The in vitro design does not fully replicate intraoral conditions, such as salivary flow, pH variations, and mechanical wear, which may influence the long-term stability of tubule occlusion. Additionally, the relatively small sample size may limit generalizability. Future studies should incorporate in vivo conditions and long-term assessments to validate these findings.

Clinically, the results suggest that Er:YAG laser activation significantly enhances the dentinal tubule-occluding efficacy of bioactive glass varnish, offering a more effective and potentially longer-lasting approach for the management of dentinal hypersensitivity compared to conventional sodium fluoride varnish.

## Conclusions

Within the limitations of this in vitro study, both sodium fluoride and bioactive glass varnishes contributed to partial dentinal tubule occlusion. Laser activation with Er:YAG enhanced the surface modifications and tubule-sealing effect, as observed under SEM. The laser-activated bioglass varnish group showed the lowest mean number of open tubules, indicating the highest degree of occlusion, while sodium fluoride without laser demonstrated the least effectiveness. Statistical analysis confirmed significant differences between the laser-activated bioglass varnish and all other groups, whereas differences among the other groups were not significant.

SEM, combined with ImageJ software, provided a reliable quantitative and qualitative evaluation of dentinal surfaces. These findings suggest that both varnishes, particularly laser-activated bioglass, may be effective for reducing open dentinal tubules in vitro. Further long-term in vivo studies are warranted to assess clinical effectiveness and durability under functional conditions.
